# Demystifying COVID-19 mortality causes with interpretable data mining

**DOI:** 10.1038/s41598-024-60841-w

**Published:** 2024-05-02

**Authors:** Xinyu Qian, Zhihong Zuo, Danni Xu, Shanyun He, Conghao Zhou, Zhanwen Wang, Shucai Xie, Yongmin Zhang, Fan Wu, Feng Lyu, Lina Zhang, Zhaoxin Qian

**Affiliations:** 1 School of Computer Science and Engineering, Central South University, Changsha, Hunan China; 2 Department of Critical Care Medicine, Xiangya Hospital, Central South University, Changsha, Hunan China; 3 Department of Electrical and Computer Engineering, University of Waterloo, Waterloo, Canada

**Keywords:** Infectious diseases, Outcomes research

## Abstract

While COVID-19 becomes periodical, old individuals remain vulnerable to severe disease with high mortality. Although there have been some studies on revealing different risk factors affecting the death of COVID-19 patients, researchers rarely provide a comprehensive analysis to reveal the relationships and interactive effects of the risk factors of COVID-19 mortality, especially in the elderly. Through retrospectively including 1917 COVID-19 patients (102 were dead) admitted to Xiangya Hospital from December 2022 to March 2023, we used the association rule mining method to identify the risk factors leading causes of death among the elderly. Firstly, we used the Affinity Propagation clustering to extract key features from the dataset. Then, we applied the Apriori Algorithm to obtain 6 groups of abnormal feature combinations with significant increments in mortality rate. The results showed a relationship between the number of abnormal feature combinations and mortality rates within different groups. Patients with “C-reactive protein > 8 mg/L”, “neutrophils percentage > 75.0 %”, “lymphocytes percentage < 20%”, and “albumin < 40 g/L” have a 2$\times$ mortality rate than the basic one. When the characteristics of “D-dimer > 0.5 mg/L” and “WBC > $9.5 \times 10^9$ /L” are continuously included in this foundation, the mortality rate can be increased to 3$\times$ or 4$\times$. In addition, we also found that liver and kidney diseases significantly affect patient mortality, and the mortality rate can be as high as 100%. These findings can support auxiliary diagnosis and treatment to facilitate early intervention in patients, thereby reducing patient mortality.

## Introduction

According to the recent World Health Organization (WHO) report on SARS-CoV-2 infections, the mortality risk remains high in vulnerable populations with reinfection, especially the elderly^1,2^. Existing studies suggest deceased patients were generally older than survivors, and the proportion was higher over 60 years of age, with a 58% increase in risk associated with every 10-year increase in age^3^. As individuals aged over 60 respond to Omicron infection, a decline in immune capacity, attributable to their age, may increase the likelihood of experiencing severe inflammation^4^. In addition, the death of elderly patients may arise from the deterioration of their basic diseases, chronic diseases, and comorbidities that they had already suffered due to COVID-19 infection^3,5,6^.

Numerous studies investigated the impact of one or several indicators on the trajectory and mortality of COVID-19. It has been highlighted the relationship between COVID-19 and multi-organ damage, including effects on the heart, lung, liver, kidney, pancreas, spleen, and the progression of underlying disease^7–9^. Moreover, a study conducted in Spain showed previous admission within the last 30 days, chronic heart disease, chronic renal failure, platelet count, incubation and mechanical ventilation at intensive care unit admission and systemic steroids all comprised independent factors for in-hospital mortality in elderly patients^10^. Unfortunately, these studies are heterogeneous with different demographics, disease severity, inclusion and exclusion criteria and outcomes. Additionally, the heterogeneity of the elderly with COVID-19 was underscored previously^11^. To which extent COVID-19 patients affected by the variables is partially unknown and the characteristics of patients with higher mortality remain to be elucidated.

Recently, researchers started combining medical investigation with artificial intelligence. Machine Learning was employed to identify several significant risk factors associated with COVID-19 lethality^12,13^. Moreover, by applying survival tree analysis, a retrospective research identified several numbers of homogeneous subgroups with different risks for mortality from COVID-19^14^. However, the methods used previously, determining threshold value by machine, are unable to facilitate the identification of relationships among these risk factors. Importantly, the obtained black-box results do not always offer clear explanations. Therefore, figuring out a new strategy to understand how these factors interact and vary would increase the risk of the elder COVID-19 patients, which is helpful for clinicians.

Hence, this present study, retrospectively conducted in the middle area of China and supported by two machine learning methods, Affinity Propagation and Apriori Algorithm, basically divided patients in groups at different mortality rate and with specific risk factors. It is likely to provide scientific knowledge useful for decreased risk of elderly patients and for adopting rationale interventions to face the tasks that the pandemic may present in the future.

## Methods

### Study design

**Figure 1:**
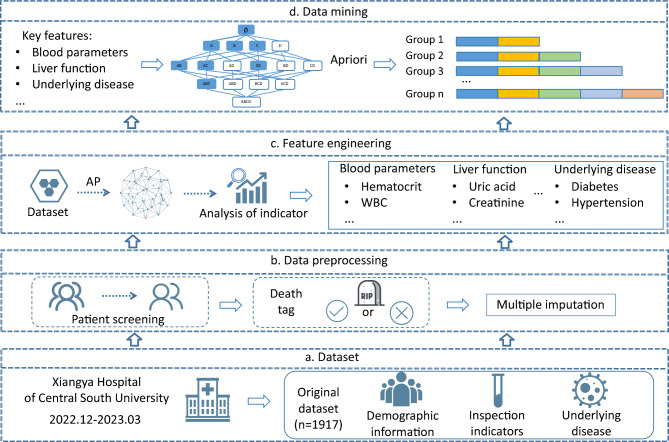
Frame diagram. The diagram illustrates a comprehensive architecture designed to analyze the combined mortality indicators of COVID-19 patients. This overall mining process can be broken down into four primary stages: data collection, data preprocessing, feature selection, and association rule mining. In the initial stage, a detailed review of the examination data of COVID-19 patients is conducted, including screening of basic disease information and demographic details, and data integrity checks. Following data preprocessing, the processed dataset was utilized to identify key features affecting COVID-19 patient mortality. This selection process leveraged both machine learning clustering techniques and established medical knowledge. Lastly, employing the association rule mining method, we derived specific mortality patterns for COVID-19 patients. These patterns not only align with existing research but also reveal fresh insights, thereby supporting clinical in diagnosis and treatment protocols.

This is a retrospective study that included all COVID-19 adult patients admitted to Xiangya Hospital, Central South University between December 2022 and March 2023. Data collected included demographic information, history of diseases, standard laboratory data including blood routine, liver and kidney function, and inflammatory markers (Fig. 1a).

Patients diagnosed with COVID-19 were included in this study. Laboratory confirmation of SARS-CoV-2 was defined as a positive result of real-time reverse transcriptase-polymerase chain reaction assay of nasal and pharyngeal swabs and, in selected cases, confirmation with reverse transcriptase-polymerase chain reaction assay from lower respiratory tract aspirates.Written informed consent was obtained from all participants when they got admitted. The investigation was approved by the Medical Ethics Committee of Xiangya Hospital, Central South University. All methods were performed in accordance with relevant guidelines and regulations. Meanwhile, we excluded patients with data completeness rates below 75% and confirmed the final outcomes of the patients. For those with missing data, we utilized multiple imputation methods for data filling (Fig. 1b).

In order to identify key features influencing mortality rate, we employed machine learning methods, Affinity Propagation (AP), to cluster patients into groups with different mortality rates. By calculating the significant differences of indicators among different groups and integrating insights from relevant research papers, we obtained the key features influencing mortality rate (Fig. 1c). The Apriori algorithm was then employed to unearth association rules related to mortality, identifying feature combinations that exhibited significantly higher mortality rates compared to the baseline and accounted for a substantial portion of patient fatalities (Fig. 1d).

### Data preprocessing

Patients with their data completeness rates below 75% were discarded. Additionally, we employed multiple imputations using chained equations to handle missing data above 75% completeness.To ensure data integrity, we excluded patients who contracted the virus after admission, including patients only with COVID-19 at admission. Furthermore, we categorized deceased patients into two groups: patients passed away during hospitalization and patients succumbed after treatment withdrawal and discharge. The latter group underwent further scrutiny, with patients discharged within 1 day following ICU discharge or still under invasive intubation evaluated for potential treatment abandonment leading to post-discharge mortality. The final patient outcomes were cross-verified with medical records to ensure accurate death labels.

### Data filling

The dataset encompassed both numerical and binary features. Since there were no missing values in binary features, our focus was on completing the numerical features. We applied multiple imputations by chained equations to fill in missing numerical data points in cases where less than 25% of a patient’s numerical features were missing. To ensure the randomness of generated data, we created five datasets for missing values. Subsequently, we averaged the missing values across these five datasets to obtain the final filled dataset.

### AP clustering and feature selection

Due to significant differences found in almost all indicators between surviving and deceased patients, it is challenging to select indicators that make a significant contribution to mortality through significance analysis alone. Therefore, we used clustering algorithms to divide patients into clusters with different mortality rates, and further select indicators that contribute to mortality rates through analysis of these clusters. The AP clustering algorithm used in this paper is a clustering algorithm based on information transmission between data points. It does not need to specify the number of clusters in advance, and the algorithm will automatically cluster patients with similar characteristics. We normalized all laboratory indicators and age, totaling 46 features, as input for AP and set the preference parameter of the AP algorithm to − 1300, which yielded four distinct classes with significant differences in mortality rates. Then, we conducted significance analysis and expert physician evaluation among these four groups to identify indicators influencing mortality rates.

### Apriori algorithm

To identify abnormal feature combinations associated with substantial increases in mortality, we used the Apriori algorithm, a association rule mining technique relying on key metrics such as support, confidence, and lift. The classic example of beer and diapers can illustrate data association rule mining, where individuals who purchased beer are also likely to purchase diapers. Similarly, in our context, we considered patients’ characteristics as “items like beer” such as “WBC > $9.5 \times 10^9$ /L” and “Cancer”. Each patient was then associated with either the item of “deceased” or “survivor” based on their individual outcomes as “diapers”. By applying the mined association rule, we aim to identify features which were associated with an increased occurrence of the “death” item, for identifying the factors affecting the mortality rate across COVID-19 patients.

In this study, support, confidence, and lift metrics correspond to the proportion of deaths, the mortality rate within each group, and the rate of mortality improvement, respectively. Notably, if an abnormal feature combination lacks relevance to mortality, the value of lift remains at 1. Otherwise, the value of lift exceeds 1. By defining the minimum support and the minimum confidence thresholds, we identified specific abnormal feature combinations. This indicates that, when patients were stratified based on these combinations, both the number of deaths and the group mortality rate within each group exceeded the defined threshold values. The calculation formulas are as follows: assuming X is a collection of various abnormal features, $X=${$feature _{i_1},feature _{i_2},feature _{i_3}\ldots$}, $X^+$ is the collection of deaths who can fit a certain set of characteristics, $X^+=${$feature _{i_1},feature _{i_2},feature _{i_3} \ldots ,die$}, and $X^-$ denotes the collection of surviving patients who can fit a certain set of characteristics, $X^-=${$feature _{i_1},feature _{i_2},feature _{i_3} \ldots ,survive$}.

1 $$\begin{aligned} {\text{ Support(the } \text{ proportion } \text{ of } \text{ deaths) } }= & {} \frac{X^{+}}{\text{ all } \text{ deaths } } \ge \text{ threshold } \end{aligned}$$

2 $$\begin{aligned} {\text{ Confidence(the } \text{ mortality } \text{ rate } \text{ within } \text{ the } \text{ group) }}= & {} \frac{X^{+}}{X^{+}+X^{-}}=\frac{X^{+}}{X} \ge \text{ threshold } \end{aligned}$$

3 $$\begin{aligned} \text {Lift}= & {} \frac{(P(X^+))}{(P(X)*P(Death))}>1 \end{aligned}$$

To obtain association rule mining related to mortality rates, we set different thresholds to generate groups of various severity. Thresholds are determined by data sample size, baseline mortality rate and clinician opinions. Firstly, we set the support as twice the baseline mortality rate and the threshold for the proportion of deaths to be over 80%, with the condition that the lift needs to be greater than 1, and resulted in Group 1. Subsequently, we progressively increased the Support threshold to three times, four times, six times, seven times, and eight times the baseline mortality rate, while appropriately lowering the threshold for the proportion of deaths to 60%, 40%, 20%, 10%, and 5%, respectively, to obtain patient groups with higher mortality rates but smaller proportions (Group 2 to Group 6). To explore the relationship between mortality rates and underlying conditions, we further reduced the threshold for the proportion of deaths to 2.5% and clustered Group 7. When exploring the relationship between underlying conditions and patient mortality rates independently, due to the relatively small proportion of patients with underlying conditions, we lowered the proportion of deceased patients to 20%, 10%, 5%, and 2.5%. Meanwhile, the support still needed to be greater than the baseline mortality rate, and the lift needed to be greater than 1.

## Results

Firstly, we divided the processed patient data into two groups: deceased and non-deceased, and conducted a significance analysis in 46 indicators. Significant disparities were observed in almost fundamental indicators (Appendix Table 1). Therefore, we cannot pinpoint the specific key indicators influencing mortality through this approach. Then, we utilized the AP clustering algorithm to achieve a more refined patient classification, resulting presented in Table 1. The clustering results yielded four classes of patients with significantly different mortality rates. Subsequently, we conducted a significance analysis of the 46 indicators for these four groups of patients to obtain the key indicators affecting their mortality rates. Based on the evaluation of these indicators, two experienced physician further evaluated these indicators and confirmed that patients in each of the four groups exhibited abnormalities in a total of 10 indicators: C-reactive protein, hematocrit, white blood cell count, hemoglobin, lymphocyte differential count, lymphocyte percentage, uric acid, creatinine, and D-dimer . Building upon previous research, we incorporated additional features such as albumin, total bilirubin, neutrophils, activated partial thromboplastin time, and underlying diseases such as pulmonary diseases, renal function diseases and cancer. We established a set of 25 features potentially influencing the probability of death in COVID-19 patients.

**Table 1:** Differences in four groups of indicators.

Indicator	Class1	Class2	Class3	Class4	P-value
C-reactive protein (mg/L)	20.21 ± 29	57.14 ± 58	48.54 ± 51	116.02 ± 82	< 0.0001
Hematocrit (%)	37.22 ± 5	37.59 ± 5	30.73 ± 6	30.2 ± 7	< 0.0001
White blood cells ($10^9$/L)	5.82 ± 3	9.14 ± 4	5.09 ± 2	11.15 ± 8	< 0.0001
Hemoglobin (g/L)	122.17 ± 18	124.13 ± 17	102.2 ± 20	98.24 ± 24	< 0.0001
Lymphocyte count ($10^9$/L)	1.62 ± 2	0.89 ± 1	0.75 ± 0	0.7 ± 1	< 0.0001
Percentage of lymphocytes (%)	27.33 ± 12	10.81 ± 6	17.13 ± 11	7.06 ± 6	< 0.0001
Uric Acid (umol/L)	285.79 ± 103	275.17 ± 116	269.52 ± 116	460.78 ± 194	<0.0001
Creatinine (umol/L)	75.45 ± 61	85.24 ± 53	99.88 ± 122	427.52 ± 381	< 0.0001
D-dimer (mg/L)	0.38 ± 1	0.63 ± 1	0.75 ± 1	4.05 ± 5	< 0.0001
Age	59.82 ± 22	68.04 ± 18	72.26 ± 16	69.05 ± 16	< 0.0001
Death rate (%)	0.01 ± 0	0.09 ± 0	0.05 ± 0	0.31 ± 0	< 0.0001

Data are presented as mean ± SD, and P values were calculated with the use of the Kruskal–Wallis test.

### Data mining results

We initiated association rule data mining analysis by Apriori Algorithm on the 25 features that might impact the mortality probability of COVID-19 patients. The mining results are detailed in Table 2. According to the results of association rule mining, we identified six distinct groups characterized by abnormal feature combinations associated with a substantial increase in mortality rate. During the analysis and observation, we noticed that the abnormal features of patients in Group 2–6 were gradually added based on Group1. As these features were added, the mortality rate of patients within these groups significantly increased. Meanwhile, we also calculated the P-values for each group of features to validate the effectiveness of our grouping (Appendix Table 3). Since each group contains the same first four indicators, no comparisons were made for these four in Table 2. In Table 2, the P-value for D-dimer was obtained from the significance analysis between Group 1 and Group 2, as these two groups exhibit differences in this variable. Similarly, WBC was obtained from the comparison between Group 2 and Group 3. Age, Lymphocyte Count, and Hematocrit were obtained from the comparison between Group 3 and Group 4, and so forth.

**Table 2:** Six groups categorized by indicators.

Indicator	Group1	Group2	Group3	Group4	Group5	Group6	P-value
C-reactive protein $>8$ mg/L	$\surd$	$\surd$	$\surd$	$\surd$	$\surd$	$\surd$	
Neutrophils percentage $>75.0$ %	$\surd$	$\surd$	$\surd$	$\surd$	$\surd$	$\surd$	
Lymphocytes percentage $<20$ %	$\surd$	$\surd$	$\surd$	$\surd$	$\surd$	$\surd$	
Albumin $<40$ g/L	$\surd$	$\surd$	$\surd$	$\surd$	$\surd$	$\surd$	
D-dimer $>0.5$ mg/L		$\surd$	$\surd$	$\surd$	$\surd$	$\surd$	< 0.0001
White blood cells $>9.5 \times 10^9$ /L			$\surd$	$\surd$	$\surd$	$\surd$	< 0.0001
Age $>70$				$\surd$	$\surd$	$\surd$	<0.0001
Age $>80$						$\surd$	0.018
Lymphocyte count $<1.1 \times 10^9$ /L				$\surd$	$\surd$	$\surd$	<0.0001
Hematocrit $<40$ %				$\surd$	$\surd$	$\surd$	0.251
Creatinine $>111$ umol/L					$\surd$	$\surd$	<0.0001
Uric Acid $>428.0$ umol/L						$\surd$	0.0007
Hemoglobin $<130$ g/L						$\surd$	0.204
Lift	2.1	3.2	4.2	5.7	7.9	8.5	
proportion of deaths	84 %	65 %	44 %	26 %	13 %	5 %	
Mortality within the group	17.30 %	27.10 %	35.40 %	48.20 %	66.70 %	71.40 %	

In order to investigate why features of underlying conditions were not present in the results (Group 1 to Group 6), we removed laboratory indicator variables to independently explore the impact of underlying conditions on patient mortality rates. Due to the relatively small proportion of patients with underlying conditions, we lowered the threshold proportion of deceased patients to 20%, 10%, 5%, and 2.5%. Meanwhile, the support still needed to be greater than the baseline mortality rate, and the lift needed to be greater than 1. We aimed to identify combinations of underlying conditions associated with increased mortality rates.

**Table 3:** The results of the significance t-test for IGD on the training set.

Feature	Age > 60	Age > 70	Age > 80	Cancer	PD	RFD	LFD	Proportion	Death rate	Lift
Category6	$\surd$	$\surd$	$\surd$	$\surd$				8%	27.60%	3.3
Category10	$\surd$	$\surd$	$\surd$		$\surd$			10%	11.80%	1.4
Category23						$\surd$		25%	16.60%	2
Category27							$\surd$	10%	14.50%	1.7
Category36	$\surd$					$\surd$	$\surd$	6.00%	54.50%	6.5
Category38	$\surd$	$\surd$	$\surd$			$\surd$	$\surd$	3.50%	57.10%	6.8

*PD* Pulmonary diseases, *RFD* Renal function diseases, *LFD* Liver function diseases.

As depicted in the Table 3, (additional details available in Appendix Table 2), we observed that different combinations of underlying diseases yielded different effects on mortality. For the same set of underlying diseases, a gradual increase in mortality is correlated with the advancing age of patients. Meanwhile, most combinations of primary disease characteristics, such as cancer and lung diseases, exhibited a lower impact on mortality compared with the combinations of abnormal indicator characteristics. Notably, liver and kidney diseases had a more significant impact on mortality than that of other underlying diseases, with the mortality rate escalated further when patients had both liver and kidney diseases. However, the proportion of these deceased patients is relatively small, which had previously been excluded from the association rule mining due to lower patient proportion thresholds. Consequently, we adjusted the threshold of deceased patient proportion to 2.5% and re-mined all features to find abnormal patient feature combinations involving underlying diseases and laboratory indicators. This finding, identified as Group 7, encompasses C-reactive protein $>8$ mg/L, neutrophils percentage $>75.0$%, lymphocytes percentage $<20$%, albumin $<40$ g/L, D-dimer $>0.5$ mg/L, WBC $>9.5\times$/L, Age $>80$, lymphocyte count $<1.1 \times 10^9$/L, hCT $<40$%, creatinine $>111$ umol/L, Uric Acid $>428.0$ umol/L, hemoglobin $<13.g$/L, totaling 12 indicators. Due to the limited size of Group 7, additional analysis is detailed in the Appendix.

**Figure 2:**
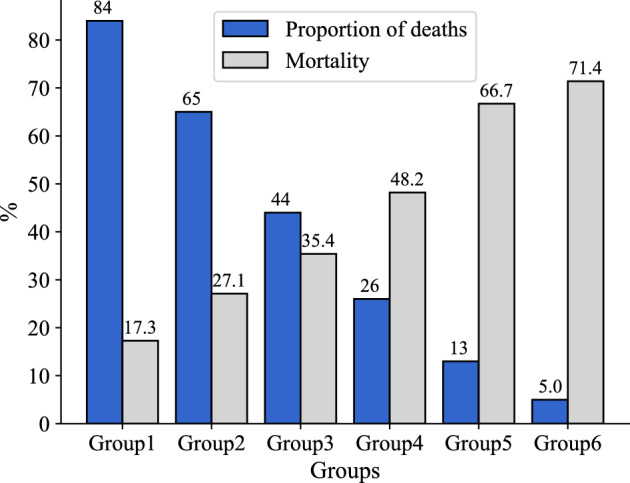
The mortality rate and the proportion of deaths in six groups.

We show bar plots to illustrate the proportion of deaths among patients and the mortality rate within each of the six combinations of abnormal characteristics (Fig. 2). The figure demonstrates that, with the increase of relevant features, the number of screened patients within each group exhibited a decreasing trend, while the mortality rate within the group continued to rise. This underscores the substantial impact of these selected indicators on patient mortality.

**Figure 3:**
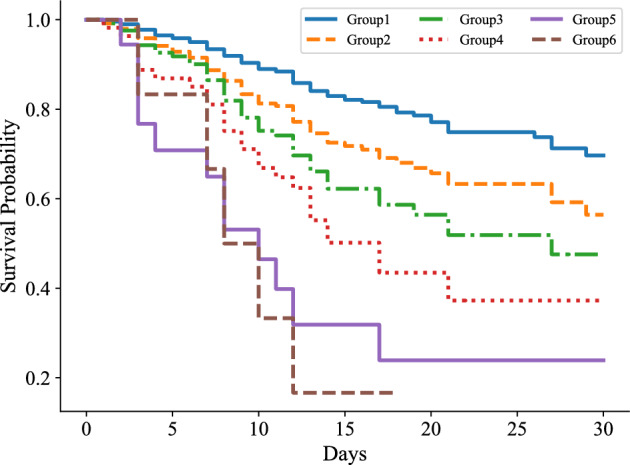
Survival curves of six groups.

We also plotted survival curves for six groups (Fig. 3). Notably, the rate of decline in the probability of survival over time varied considerably among the groups. As we progressed from group 1 to group 6, the risk of death and the rate of deterioration increased gradually with the accumulation of more abnormal indicators.

## Discussion

In this study, we gathered data of 1917 COVID-19 patients from Xiangya Hospital, Central South University. To investigate the risk factors influencing mortality, we initially employed the Affinity Propagation clustering method, to identify potentially significant indicators. Subsequently, we employed the Apriori algorithm to mine association combinations influencing mortality. Present analysis revealed patients with “C-reactive protein > 8 mg/L”, “neutrophils percentage > 75.0%”, “lymphocytes percentage < 20%” and “albumin <40 g/L” exhibited a twice higher mortality rate than the baseline. Furthermore, the presence of “D-dimer > 0.5 mg/L” in conjunction with the aforementioned characteristics was associated with a mortality rate three times higher than the baseline. Through these feature combinations, we categorized the patients into seven groups, each showing an escalating trend in mortality as the number of relevant features increased.

Association rule mining generated a basic group characterized by high-level CRP, high neutrophils, low lymphocytes, and low albumin. Basically, patients in this group were in the hyperinflammation and malnutrition status. The elevated level of CRP indicates the presence of infection, especially bacterial pathogen or inflammatory diseases, particularly cardiovascular diseases^15^. The increase of CRP has been reported in several viral infections, such as H1N1^16^ and recent SARS-CoV2^17–19^. Patients with elevated CRP level concentrations at the time of initial presentation were more likely to be associated with critical illness, and in-hospital mortality during subsequent hospital stays compared with those with lower initial measurements. Patients with the highest CRP level are the worst in this group^20^. This study clearly demonstrated the relationship between CRP level and COVID-19 severity. Additionally, both observational study and meta-analysis supported CRP as a prognostic factor in assessing disease lethality for COVID-19 patients^21–23^. A retrospective study showed CRP measured both on admission and during the course of the disease in patients with COVID-19 was helpful for guiding therapy^24^.

Compared with patients without disease progression, those with disease progression presented persistently low lymphocyte counts and elevated CRP levels^25^. When CRP was combined with lymphocytes, it turned out to be a significant predictive factor in poor short-term clinical outcomes of SARS-CoV-2 BA.2.2 patients^26^. However, a multicenter study did not support the lymphocyte-to-CRP ratio as an accurate factor to predict disease severity or mortality^27^, which indicated other factors should be coupled to achieve a more reliable prediction model. Moreover, the negative correlation between lymphocyte subset and CRP could be a useful tool to predict patients’ responses to therapy, particularly for patients with relatively lower WBC^28^. Our study combined high CRP, high neutrophils, low lymphocytes, and low albumin as a group and defined a population at high risk of death, providing a new predictive strategy for COVID-19.

When further adding the level of D-dimer into evaluation, we identified a subset of the basic group, group2, with a higher mortality rate. D-dimer is a blood protein, released upon the breakdown of clots. It was shown that serum D-dimer concentrations in severe COVID-19 patients were significantly higher than those presented with non-severe forms^29^. Moreover, the variation in the level of D-dimer was positively correlated with the prognosis of COVID-19 regardless of whether patients had cardiovascular diseases^30^. Irrespective of venous thromboembolism, the level of D-dimer at hospital admission for COVID-19 was associated with in-hospital mortality^31^. The D-dimer level not only was closely related to COVID-19 severity but also might influence the likelihood of rapid onset of organ injury after admission^32^. International guidelines suggest an age-adjusted D-dimer test threshold, rather than a simple D-dimer, must be considered for people older than 50 years^33^. In our model, based on a high level of CRP, increased neutrophil, low lymphocytes, and low albumin, patients with a high level of D-dimer were with a higher mortality, indicating that the measurement of D-dimer is helpful for COVID-19 prediction, which is consistent with previous findings.

Our analysis showed group 4 had a higher mortality, in which patients are older than 70. Actually, individuals of any age are susceptible to SARS-CoV2, but older individuals are more vulnerable and suffering from an aggressive form of COVID-19. The age distribution of deaths in younger age groups (less than 65 years of age) was previously shown very consistent across different settings, whereas the mortality in individuals of age over 65 is significantly heterogeneous^34^. Additionally, studies have indicated that longer viral shedding was tightly associated with advanced age^35^. Essential explanations for this scenario would be the dynamic remodeling of immune response. As age advances, the responses of T cells and B cells decrease, and the dysfunction of innate immunity is observed as well, which is called immunosenescence^36^. Innate immunity, as the first defense, protects bodies against pathogens by secreting interferon to achieve an antimicrobial state to limit viral replication^37^. However, due to immunosenescence characterized by delayed IFN reaction, pathogens could escape from innate immunity at the early stage of infection, and older individuals would be at high risk of severe infection^38^. Delayed and insufficient activation of innate immunity contributed to weakened adaptive immunity, which is also a part of immunosenescence. All mentioned above postponed the clearance of virus and lead to dysregulated immune response, thereby causing the cytokine storm and severe conditions in older patients^39^. Most older individuals develop inflammaging, a condition characterized by elevated levels of blood inflammatory markers that carries a high susceptibility to chronic morbidity, disability, frailty, and premature death^40^. Under general circumstances, inflammaging functions as a protective mechanism in the older population. When balance is disturbed, the dysregulation of anti-inflammatory elements would bring about an excessive inflammatory response^39,41^. This high inflammatory status is consistent with high level CRP, as shown above, and the high production of IL-6 and tumor necrosis factor-a^42,43^.

As shown, patients with a history of organ dysfunction have a higher risk of developing severe COVID-19, which in turn can promote acute multiorgan injury^44,45^. In this model that includes common comorbidities, liver and renal dysfunction are identified as potential factors for predicting high mortality. Nevertheless, there were only 2 patients in group 7 and the mortality rate within group is 100%, which seems illogical and weakens the confidence of this conclusion. As known, patients with chronic kidney disease were more inclined to present abnormal uric acid and creatinine, so it is difficult to figure out which one was dominant in this analysis. Liver function tests were found to be an independent predictor of death or transfer to ICU in COVID-19^46,47^. COVID-19 related mortality is about ten times higher than that of chronic kidney disease patients without COVID-19^48^. It is speculated that COVID-19 is influenced by two mechanisms. First, compared to other organ injuries, liver and renal dysfunction were more prevalent among the population, especially individuals of advanced age, who were at a relatively malnutrition and suppressed immune status^49^. Second, it has been found that patients with severe COVID-19 commonly had higher levels of inflammatory factors than those with non-severe forms, indicating that a cytokine storm was associated with disease severity^50^. Individuals with chronic diseases have been in a proinflammatory state with impaired immunity thus, are vulnerable to severe COVID-19. Limited by the sample size, group 7 requires more prospective studies and large cohort studies to get verified.

Decreased hemoglobin levels can aggravate cell hypoxia and damage to tissue and organs, and lung injury causes inflammation and infiltration, thereby affecting body oxygenation. A retrospective cohort study implied that anemia was an independent risk factor for COVID-19^51^, which was further verified by a prospective study in Iran^52^. Furthermore, hemoglobin of < 12 g/dL for females and < 13 g/dL for males were significantly associated with the risk of mortality^53^. However, in present study, hemoglobin < 130 g/L appeared non-significant in dividing group, which might attribute to the gender ratio that was not clarified.

There are emerging studies using machine learning to evaluate mortality risk of COVID-19. Random forest model^12^ and survival tree analysis^14^ were ultilized in several database investigation, and these methods found out significant indicators for predicting the outcome of COVID-19^54^. Extreme gradient boosting technique and neural network^55^were then used to further improve the prediction model established before^11^. However, these models were mainly generated by machine, so they failed to reveal the interaction of indicators and explain why these indicators could affect mortality. Different from these previous studies, we utilized two machine learning methods: AP Clustering and Apriori Algorithm. Before machine learning, we set the threshold from the clinicians’ perspective, then association rule mining was applied to recognize similar association between indicators and outcome. In this way, sample error could be minimized and the interpretability could be maximized.

## Conclusions

The current study has effectively summarized the medical characteristics of the patients and has identified seven distinct groups at different risk of mortality. Healthcare providers should heighten their awareness of features such as age, CRP, lymphocyte and neutrophil levels, albumin, D-dimer, hemoglobin, and comorbidities. Clinicians can identify individuals and implement tailored treatment strategies, ultimately facilitating reducing the incidence of severe COVID-19 cases and overall mortality.

### Advantages and limitations

Advantages: For parameters selection, we used AP clustering and significance analysis rather than LASSO and survival tree analysis. Patients were clustered into different groups with various mortality rates, and then we investigated which indicators contributed the differences in mortality rates. This method is more reliable than directly using results from models. Then, we further explored how the combinations of indicators interactively influenced mortality rates. We set thresholds for indicators based on medical standards and used the association rule analysis algorithm, Apriori, to discover which abnormal combinations of indicators significantly affected mortality rates. Compared to methods such as XGBoost and random forest models, which derive results based on thresholds determined by the model, our approach is better at reducing biases and improving interpretability.

Limitations: It is worth noting that the majority of our patients originated from the middle area of China. This might introduce certain demographic disparities and statistical biases into our analysis. Meanwhile, limited by the sample size, group 7 requires large cohort studies or retrospective investigation to be verified.

### Supplementary Information

Supplementary Information.

## Data Availability

The datasets generated during and/or analyzed during the current study are not publicly available due to the confidential policy of National Health Commission of China, but are available from the corresponding author Lina Zhang upon reasonable request.
