# Supplementary material for: Demystifying COVID-19 mortality causes with interpretable data mining
Source: Sci Rep. 2024 May 2;14:10076. doi: 10.1038/s41598-024-60841-w (PMC11066015; doi:10.1038/s41598-024-60841-w)
Supplement: Supplementary file 1 — Supplementary Information. [file 41598_2024_60841_MOESM1_ESM.pdf]

Supplementary Information

Table 1: Comparison of Dead/Surviving Patients.

Feature	All (n=1181)	Missing rate	Survival (n=1079)	Death (n=102)	P-value
C-reactive protein	53.96±60	0.30	49.38±56	102.49±78	<0.05
Percentage of eosinophils	0.92±2	0	0.97±2	0.37±1	<0.05
Percentage of basophils	0.27±0	0	0.28±0	0.21±0	<0.05
Red cell distribution width	13.97±2	0.11	13.91±2	14.54±2	<0.05
Platelet hematocrit	0.19±0	0.02	0.2±0	0.15±0	<0.05
Mean platelet volume	9.78±1	0.02	9.72±1	10.4±1	<0.05
hCT	35.15±7	0	35.24±6	34.24±7	0.060807832
White blood cells	7.49±5	0	7.2±5	10.55±5	<0.05
RBC	3.86±1	0	3.87±1	3.69±1	<0.05
Hemoglobin	115.8±22	0	116.22±22	111.27±25	<0.05
MCV	91.64±7	0	91.51±7	93.04±7	<0.05
Mean hemoglobin content	30.21±3	0	30.21±3	30.24±3	0.405401466
MCH	329.43±15	0	329.83±15	325.19±14	<0.05
Blood platelet count	204.61±107	0.002	209.99±108	147.77±76	<0.05
Neutrophil count	5.75±4	0	5.41±4	9.35±5	<0.05
Monocyte count	0.61±1	0	0.62±1	0.49±0	<0.05
Eosinophil count	0.06±0	0	0.06±0	0.04±0	<0.05
Basophil count	0.02±0	0	0.02±0	0.02±0	0.906546212
Lymphocyte count	1.06±2	0	1.1±2	0.64±1	<0.05
Percentage of lymphocytes	16.64±12	0	17.55±12	6.99±7	<0.05
Percentage of monocytes	8.69±6	0	9.0±6	5.4±4	<0.05
Percentage of neutrophilic granulocyte	73.48±16	0	72.2±15	87.05±10	<0.05
Width of platelet volume distribution	14.56±3	0.02	14.46±3	15.59±3	<0.05
TP	61.57±8	0.01	61.93±7	57.73±8	<0.05
Albumin	33.54±5	0.01	33.97±5	29.0±5	<0.05
Globulin	28.05±6	0.01	27.97±5	28.92±6	0.068769811
Total bilirubin	12.57±18	0.01	12.26±18	15.85±15	<0.05
Direct bilirubin	5.14±11	0.01	4.89±11	7.78±10	<0.05
Total bile acid	6.23±12	0.01	6.14±12	7.23±15	0.455663095
Alanine aminotransferase	46.07±137	0.12	37.47±53	136.99±424	0.08382207

Continued on next page

Table 1 – Continued from previous page

Feature	All (n=1181)	Missing rate	Survival (n=1079)	Death (n=102)	P-value
Aspartate amino transferase	75.59±513	0.12	42.6±69	424.48±1701	<0.05
White sphere ratio	1.24±0	0.11	1.26±0	1.03±0	<0.05
Uric Acid	302.48±139	0.01	296.28±128	368.1±217	<0.05
Carbonyldiamide	9.15±8	0.01	8.41±7	17.01±12	<0.05
Creatinine	125.25±182	0.01	118.06±176	201.27±223	<0.05
Prothrombin time	12.37±3	0.10	12.2±2	14.1±6	<0.05
Activated partial thromboplastin time	29.15±8	0.10	28.73±7	33.64±15	<0.05
Thrombin time	17.37±11	0.10	16.99±8	21.4±25	<0.05
Fibrinogen	4.25±2	0.10	4.24±2	4.34±2	0.765143603
Percentage of prothrombin	91.1±17	0.10	92.31±16	78.37±21	<0.05
International standard ratio	1.06±0	0.10	1.05±0	1.23±1	<0.05
Plasma fibrinogen (fibrinogen) degradation products	9.08±18	0.20	7.46±15	26.22±31	<0.05
Determination of antithrombin III antigen in plasma	293.0±76	0.20	296.55±77	255.45±50	<0.05
Plasma plasminogen antigen measurement	270.92±28	0.10	271.02±28	269.85±34	0.712566673
D-dimer	0.93±2	0.10	0.74±2	2.95±4	<0.05
Length of stay	11.84±9	0	11.92±9	10.95±9	0.141411026
Length of ICU stay	7.51±6	0	6.89±6	8.1±7	0.252577415
ICU Admission	156/1181(13.2%)	0	76/1079(7.0%)	80/102(78.4%)	
Age	66.78±19	0	65.99±19	75.17±15	<0.05
60	331/1181(28.0%)		320/1079(29.6%)	11/102(10.8%)	
60-69	231/1181(19.6%)		214/1079(19.9%)	17/102(16.6%)	
70-79	282/1181(23.9%)		255/1079(23.6%)	27/102(26.5%)	
>80	337/1181(28.5%)		290/1079(26.9%)	47/102(46.1%)	
Gender					<0.05
Male	722/1181(61.1%)		644/1079(59.7%)	78/102(76.5%)	
Female	459/1181(38.9%)		435/1079(40.3%)	24/102(23.5%)	
Diabetes	341/1181(28.9%)		303/1079(28.1%)	38/102(37.3%)	0.065796573
Hypertensive	570/1181(48.3%)		519/1079(48.1%)	51/102(50%)	0.792253351
Coronary heart disease	323/1181(27.3%)		288/1079(26.7%)	35/102(34.3%)	0.124895038
Lung diseases	212/1181(18.0%)		189/1079(17.5%)	23/102(22.5%)	0.258060253
Heart failure	50/1181(4.2%)		46/1079(4.3%)	4/102(3.9%)	1

Continued on next page

Table 1 – Continued from previous page

Feature	All (n=1181)	Missing rate	Survival (n=1079)	Death (n=102)	P-value
Myocardial infarction	55/1181(4.7%)		51/1079(4.7%)	4/102(3.9%)	0.902103515
Cerebral infarction diseases	135/1181(11.4%)		120/1079(11.1%)	15/102(14.7%)	0.355112726
Arrhythmia	72/1181(6.1%)		66/1079(6.1%)	6/102(5.9%)	1
Cancer	172/1181(14.6%)		158/1079(14.6%)	14/102(13.7%)	0.916921369
Renal function diseases	139/1181(11.8%)		116/1079(10.8%)	23/102(22.5%)	<0.05
Liver function diseases	64/1181(5.4%)		55/1079(5.1%)	9/102(8.8%)	0.173802538

Note: Pulmonary diseases include chronic obstructive pulmonary disease, emphysema, and chronic bronchitis. Cancer includes various malignant solid tumors, leukemia, and lymphoma. Renal function diseases include renal insufficiency, renal failure (acute and chronic); Liver function diseases include hepatitis, cirrhosis, and hepatic insufficiency.

Table 2: Table of Disease Analysis Results.

Feature	Age60	Age70	Age80	Cancer	PD	CHD	Diabetes	RFD	LFD	CI	HTN	Proportion	Death rate	Lift
Category1	✓											88%	10.40%	1.2
Category2	✓	✓										71%	11.80%	1.4
Category3	✓	✓	✓									45%	14.10%	1.7
Category4	✓			✓								13%	14.40%	1.7
Category5	✓	✓		✓								10%	16.70%	2
Category6	✓	✓	✓	✓								8%	27.60%	3.3
Category7					✓							24%	10.60%	1.3
Category8	✓				✓							23%	11.70%	1.4
Category9	✓	✓			✓							20%	12.20%	1.5
Category10	✓	✓	✓		✓							10%	11.80%	1.4
Category11						✓						36%	10.40%	1.2
Category12	✓					✓						32%	10.80%	1.3
Category13	✓	✓				✓						31%	12.20%	1.4
Category14	✓	✓	✓			✓						18%	13.80%	1.6
Category15					✓	✓						14%	17.30%	2.1
Category16	✓				✓	✓						13%	17.80%	2.1
Category17	✓	✓			✓	✓						11%	17.50%	2.1
Category18	✓	✓	✓		✓	✓						7%	17.10%	2
Category19							✓					41%	11%	1.3
Category20	✓						✓					38%	13%	1.6
Category21	✓	✓					✓					31%	14.40%	1.7
Category22	✓	✓	✓				✓					17%	16.50%	2
Category23								✓				25%	16.60%	2
Category24	✓							✓				19%	19.00%	2.3
Category25	✓	✓						✓				14%	17.90%	2
Category26	✓	✓	✓					✓				8%	20.00%	2.4
Category27									✓			10%	14.50%	1.7
Category28	✓								✓			8%	19.00%	2.3
Category29	✓	✓							✓			6%	21.40%	2.5
Category30	✓	✓	✓						✓			5%	27.80%	3.3
Category31										✓		14%	10.30%	1.2
Category32	✓									✓		13%	10.90%	1.3
Category33	✓	✓								✓		10%	10.50%	1.2
Category34	✓	✓	✓							✓		8%	16.00%	1.9
Category35								✓	✓			8.80%	42.10%	5
Category36	✓							✓	✓			6.00%	54.50%	6.5
Category37	✓	✓						✓	✓			3.80%	44.40%	5.3
Category38	✓	✓	✓					✓	✓			3.50%	57.10%	6.8
Category39								✓	✓		✓	6.30%	54.30%	6.5
Category40	✓							✓	✓		✓	3.50%	57.10%	6.8
Category41	✓	✓						✓	✓		✓	2.00%	50.00%	5.9
Category42	✓	✓	✓					✓	✓		✓	2.00%	75.00%	8.9

Note: Pulmonary diseases include chronic obstructive pulmonary disease, emphysema, and chronic bronchitis. Cancer includes various malignant solid tumors, leukemia, and lymphoma. Renal function diseases include renal insufficiency, renal failure (acute and chronic); Liver function diseases include hepatitis, cirrhosis, and hepatic insufficiency.

Table 3: Comparison of Six Groups

Indicator	Group1	Group2	Group3	Group4	Group5	Group6	Group1-2 p	Group2-3 p	Group3-4 p	Group4-5 p	Group5-6 p
C-reactive protein	78.04±60	85.04±66	116.47±77	76.35±47	115.79±76	139.06±96	0.441258683	0.002302015	0.00988381	0.140547072	0.791415284
Neutrophils Percentage	84.86±6	86.75±6	86.62±6	90.5±5	92.4±3	92.09±3	0.004497486	0.87636292	0.00065754	0.401600516	0.374937154
Lymphocytes Percentage	8.54±4	7.13±4	7.33±4	4.26±3	3.81±2	3.19±1	0.003563831	0.860104779	0.000148889	0.666122597	0.467125056
Albumin	31.5±4	28.97±4	29.96±4	27.88±3	25.95±3	28.04±3	9.30759E-08	0.173713039	0.016686701	0.086535946	0.246292107
D-dimer	0.23±0	2.46±3	3.7±5	2.88±3	5.65±6	3.64±3	1.78631E-54	0.14595901	0.666428842	0.265173195	0.658999497
White blood cells	8.01±4	6.73±2	15.74±8	13.59±3	12.41±2	16.89±6	0.056911869	1.90906E-30	0.613492642	0.450199612	0.055555556
Age	69.75±17	71.78±16	62.21±19	83.0±7	81.18±7	89.0±4	0.325246218	0.000298424	2.1175E-10	0.449353394	0.018354531
Lymphocyte Count	0.61±0	0.47±0	1.09±1	0.53±0	0.47±0	0.51±0	0.000746684	2.5818E-12	2.71092E-06	0.611656827	0.646210015
Hematocrit	34.04±6	32.08±7	34.56±7	33.08±5	33.53±4	30.23±7	0.005416983	0.016651711	0.25079647	0.961772431	0.276628881
Creatinine	165.24±229	163.05±207	156.5±167	74.98±20	245.71±170	297.44±159	0.939445928	0.942925824	0.045062395	5.84505E-07	0.210910005
Uric Acid	299.7±138	302.86±173	363.74±230	246.67±94	347.62±96	570.51±100	0.639212001	0.14636428	0.017448171	0.00388369	0.000754148
Hemoglobin	112.73±22	105.52±25	112.5±24	108.08±17	110.18±13	96.86±24	0.003369691	0.058858376	0.379740307	0.904569442	0.204354023

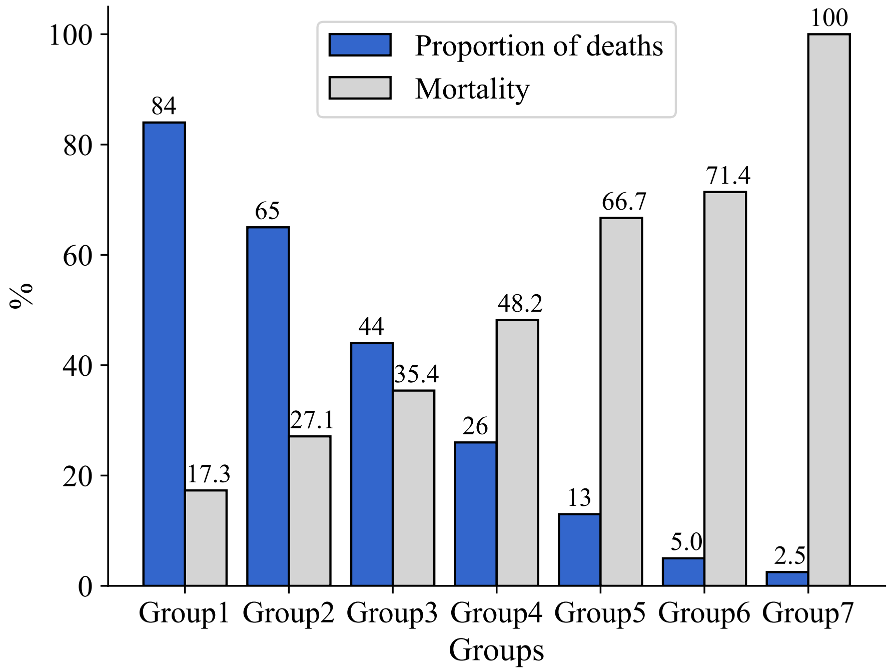

Figure 1: The mortality rate and the proportion of deaths in seven groups

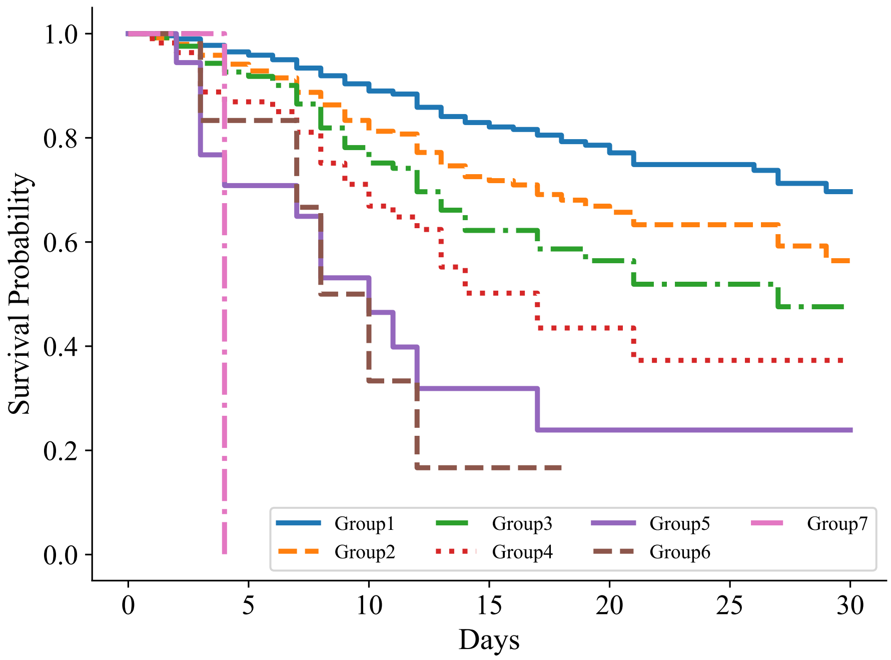

Figure 2: Survival curves of seven groups
